# Review of the genus *Stephanospathius* Belokobylskij, 1992 (Hymenoptera, Braconidae), with discussion of their tribal position

**DOI:** 10.3897/zookeys.118.914

**Published:** 2011-07-13

**Authors:** Sergey A. Belokobylskij

**Affiliations:** 1 Zoological Institute, Russian Academy of Sciences, St. Petersburg 199034, Russia; Museum and Institute of Zoology PAN, Wilcza 64, 00–679 Warsaw, Poland

**Keywords:** Braconidae, Stephaniscini, Leptospathiini, new species, new name, redescription, key to genera and species

## Abstract

A review of the Afrotropical genus *Stephanospathius* Belokobylskij is provided. A new species *Stephanospathius benoiti* **sp. n.** from the Republic of Congo, and the male and, for the first time, the female of *Stephanospathius ornatipes* (Kieffer) are described. A discussion of the status and composition of the tribe Stephaniscini is given and a new name for this tribe, Leptospathiini, **nom. n.**, is proposed. A key to the included genera and a key to species of *Stephanospathius* are provided.

## Introduction

The fauna of the braconid subfamily Doryctinae of the Afrotropical region had been studied for more than two centuries, but our knowledge of this group, even at generic level, was fragmentary and incomplete until recently (Belokobylskij and Quicke 1999, Belokobylskij 2005). At present, 37 doryctine genera in ten tribes are recorded in this zoogeographic region (Shenefelt and Marsh 1976, Belokobylskij 1992, 2005, Yu et al. 2005, etc.), including 17 endemic genera (*Leptorhaconotus* Granger, 1949, *Pseudodoryctes* Szepligeti, 1915, *Schlettereriella* Szépligeti, 1904, *Stephanospathius* Belokobylskij, 1992, *Ivondrovia* Shenefelt & Marsh, 1976, *Bathycentor* Saussure, 1892, *Cryptodoryctes* Belokobylskij & Quicke, 2000, *Bulbonervus* Shenefelt, 1969, *Paradoryctes* Granger, 1949, *Doryctoproctus* Belokobylskij, 2005, *Grangerdoryctes* Belokobylskij, 2005, *Odontodoryctes* Granger, 1949, *Priosphys* Enderlein, 1920, *Terate* Nixon, 1943, *Spathioplites* Fischer, 1962, *Toka* Nixon, 1943, *Afrospathius* Belokobylskij & Quicke, 2000, and *Hemispathius* Belokobylskij & Quicke, 2000). Only a single large revision of this group (as well as all Braconidae) has been provided for the fauna of Madagascar (Granger 1949), but numerous additional new taxa from this island await description.

The peculiar Afrotropical genus *Stephanospathius* Belokobylskij was described for the type species *Stenophasmus ornatipes* Kieffer from Cameroon (Belokobylskij 1992), and only a single male of this rare taxon was known until now. Additional morphological information about this genus was published as result of a study of internal sculpture and valvilli of ovipositor by Rahman et al. (1998). Only a single host is known for the members of this tribe, viz. *Schlettereriella gardneri* (Nixon) which was reared from *Oemida gahani* Distant (Cerambycidae) (Nixon 1954). Given the host range of related taxa, as well as the known host of *Schlettereriella*, it is possible to suggest that *Stephanospathius* species parasitize larvae of the family Cerambycidae and analogous xylophagous beetle groups inhabiting similar ecological conditions. A new Afrotropical species of *Stephanospathius*, as well as first recorded female of the type species of this genus, *Stephanospathius ornatipes* (Kieffer), are described below and keys to species of *Stephanospathius* and genera of the tribe Leptospathiini nom. n. (Stephaniscini nom. nudum: Wharton and Achterberg 2000, Yu et al. 2005) are provided.

## Material and methods

The terminology of morphological features, measurements and sculpturing follows Belokobylskij and Tobias (1998), the nomenclature for the wing venation follows Belokobylskij and Tobias (1998) and Belokobylskij and Maeto (2009). The following abbreviations are used: POL – postocellar line; OOL – ocular-ocellar line; Od – maximum diameter of lateral ocellus; SDEI – Senckenberg Deutsche Entomologische Institut (Müncheberg, Germany); MRAC – Musee royal de l’Afrique centrale (Tervuren, Bergium); ZISP – Zoological Institute, Russian Academy of Sciences (St. Petersburg, Russia).

## Systematic part

### Tribe Leptospathiini nom. n.

Stephaniscini Enderlein, 1912: 1 (type genus *Stephaniscus* Kieffer, 1904, junior homonym of *Stephaniscus* Haeckel, 1884), invalid name (Yu et al. 2005); Shenefelt and Marsh 1976: 1372, Fischer 1981a: 44, 1981b: 122, Belokobylskij 1992: 918.

#### Type genus.

*Leptospathius* Szepligeti, 1902.

#### Description.

Head subcubical in dorsal view, weakly transverse. Occipital carina mainly present. Malar suture absent or sometimes very shallow. Maxillary palpi very long or (in apterous form) short. Scape of antenna wide and short, without apical lobe or basal constriction. First segment of antennal flagellum usually shorter than second segment, sometimes almost equal to it, rarely longer than second one. Mesosoma elongate, sometimes weakly depressed, but strongly transformed in apterous or micropterous forms. Notauli present, usually shallow posteriorly. Prepectal carina present; postpectal carina absent. Sternaulus (precoxal suture) present or absent. Propodeum usually without delineated areas, with distinct propodeal bridge (heavy sclerites between coxal and metasomal foramens). Fore wing. Radial cell not shortened. Both radiomedial veins present. Position of recurrent vein variable. Discoidal cell anteriorly usually shortly petiolate. Parallel vein not interstitial. Brachial cell closed in distal posterior margin by brachial vein. Transverse anal veins absent. Hind wing with 4 hamuli. Medial cell large, distinctly widened towards apex. Radial cell widened apically, sometimes with transverse medial vein. Submedial cell usually short, but sometimes long. Hind wing of male without stigma-like enlargement. Sometimes wings absent or scale-shaped. Fore tarsus often long or very long. Hind coxa long, often (but not always) without basal tooth. All femora rather slender and elongate. First metasomal tergum long and narrow, much longer than its apical width, dorsope absent or very small, acrosternum distinctly or strongly elongated. Second tergum with basal subtriangular area more or less distinctly delineated by furrows, but sometimes with divergent long lateral furrows; apterous and micropterous forms without furrows. Laterotergites maximum separated in second and basal half of third tergites. In fully-winged taxa, terga behind second one entirely covered by very dense, rather short and pale setae. Ovipositor sheath long, longer than metasoma.

#### Distribution.

Afrotropical, Oriental, Australasian and Neotropical Regions.

**Contents.** Tribe includes five genera: *Austrospathius* Belokobylskij, Iqbal & Austin, 2004; *Leptospathius* Szepligeti, 1902; *Oroceguera* Seltmann & Sharkey, 2007; *Schlettereriella* Szepligeti, 1904; *Stephanospathius* Belokobylskij, 1992.

#### Diagnosis.

Leptospathiini is related to the tribe Spathiini characterised by elongate acrosternum of the first metasomal tergum. The main and unique apomorphic character of Leptospathiini is the presence of distinct and usually wide propodeal bridge (heavy sclerites between coxal and metasomal foramens); the feature is unknown in other doryctine groups (including similar subtribe Trigonophasmina of the tribe Spathiini: Belokobylskij 1992). Additional but not comprehensive diagnostic features of this tribe separating it from the tribe Spathiini are: the second tergum often with furrows, the parallel vein of fore wing always not interstitial and arising from the posterior one-third of the distal margin of brachial cell, and the propodeum usually without delineated areas.

#### Key to genera of the tribe Leptospathiini

**Table:** 

1	Apterous or micropterous forms. Ocelli absent. Second tergum without any furrows	2
–	Fully-winged forms. Ocelli present. Second tergum with two more or less distinct furrows	3
2	Propodeal tubercles distinct. First flagellar segment distinctly longer than second segment. Propodeal bridge narrow. Acrosternum of first tergum long, 0.7 times as long as tergum. Vertex and frons coarsely and densely rugose-reticulate. Australasian Region	*Austrospathius* Belokobylskij, Iqbal & Austin
–	Propodeal tubercles absent. First flagellar segment subequal or slightly shorter than second segment. Propodeal bridge wide. Acrosternum of first tergum short, 0.4 times as long as tergum. Vertex and frons smooth. Neotropical Region (Costa Rica)	*Oroceguera* Seltmann & Sharkey
3	First flagellar segment of antennae almost equal to or weakly shorter than second segment. Fore femur on inner side with numerous coarse regular oblique carinae. Hind coxa of female with distinct basoventral tooth; hind coxa of male without tooth. Sternaulus absent. Acrosternum of first tergum long, about 0.8 times as long as tergum. Recurrent vein postfurcal or interstitial. Pronope and high pronotal carinae always present. Afrotropical Region	*Stephanospathius* Belokobylskij
–	First flagellar segment of antennae distinctly shorter than second segment. Fore femur on inner side without carinae. Hind coxa of female and male without tooth. Sternaulus present. Acrosternum of first tergum short, less than 0.5 times as long as tergum. Recurrent vein antefurcal. Pronope and pronotal carinae usually absent	4
4	Frons with large lateral protuberances. Base of ocellar triangle less than its lateral sides. Lateral furrows of second metasomal tergum divergent posteriorly and touched second suture. Submedial cell of hind wing small; first abscissa of mediocubital vein 0.2–0.3 times as long as second abscissa. Radial cell of hind wing without transverse vein. Afrotropical Region	*Schlettereriella* Szepligeti
–	Frons without lateral protuberances. Base of ocellar triangle not less than its lateral sides. Lateral furrows of second metasomal tergum strongly convergent posteriorly and restricted basal subtriangular area, far spaced of second suture. Submedial cell of hind wing large; first abscissa of mediocubital vein subequal to second abscissa. Radial cell of hind wing with more or less distinct transverse vein. Oriental and Australasian Regions	*Leptospathius* Szepligeti

### Stephanospathius

Belokobylskij, 1992

http://species-id.net/wiki/Stephanospathius

Schlettereriella Belokobylskij 1992: 919, Rahman et al. 1998: 334, Yu et al. 2005.

#### Type species.

*Stenophasmus ornatipes* Kieffer, 1911.

#### Diagnosis.

This genus resembles *Schlettereriella* Szepligeti. Besides the characters, given in the key, *Stephanospathius* also differs in having the fore trochantellus dorsally with distinct transverse carina transformed to pointed flange on its inner part, the vertex and most of mesoscutum smooth, hypopygium with distinct insertion on posterior margin, and the second suture weakly curved and fine.

#### Description.

Head usually not depressed. Antennal sockets large (Fig. 2). Frons weakly concave, without median keel or furrow, laterally along eyes with distinct subparallel carinae fused anteriorly with margin of antennal socket. Ocelli arranged in obtuse anteriorly triangle (Fig. 2). Eyes glabrous, with weak emargination opposite antennal sockets. Occipital carina not joined below with hypostomal carina at short distance but situated very far from base of mandible. Malar suture very shallow or almost indistinct. Clypeus distinctly convex, with distinct lower flange; clypeal suture complete. Hypoclypeal depression rather large and round. Postgenal bridge rather wide. Maxillary palpi 6-segmented, labial palpi rather short and 4-segmented. Third segment of labial palpi not shortened, rather long. Antenna slender, long, almost filiform. Pedicel basally with rather distinct carina on dorsal side. First flagellar segment subcylindrical, weakly curved, almost as long as second segment.

**Figures 1–12.**
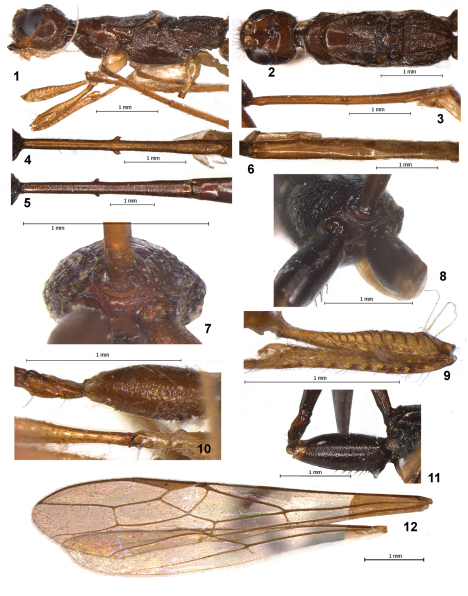
*Stephanospathius ornatipes* (Kieffer) (**1–4**, **6**, **7**, **9**, **10**, **12** – male; **5**, **8**, **11** – female) **1** head and mesosoma, lateral view **2** head and mesosoma, dorsal view **3** first tergite, lateral view **4, 5** first tergite, dorsal view **6** second-fifth tergites, dorsal view **7, 8** propodeum and propodeal bridge, posterior view **9** fore femur and tibia, inner view **10** hind coxa, trochanters and trochantellus and basal part of middle leg **11** hind coxa, trochanters and trochantellus **12** fore and hind wings.

Mesosoma more or less distinctly depressed (Figs 1, 14). Promesosoma long, wide and laterally convex in basal 0.7 and narrow in apical 0.3. Pronotal carina absent laterally, dorsally in anterior 0.3 transformed in thick flange, curved up and divided medially by rather distinct excavation (Fig. 2); before this flange developed more or less wide and deep pronope. Sides of pronotum without delineated median groove. Posterior propleural lobe long and wide. Mesonotum weakly or very weakly and gently-roundly elevated above pronotum (Figs 1, 14). Median lobe of mesonotum without median furrow and anterolateral shoulders. Notauli shallow, complete, rather wide, fused in almost middle of mesoscutum (Figs 2, 15). Prescutellar depression rather long, deep or shallow, with median carina; high lateral longitudinal wing-like flanges developed on the level of depression. Scuto-scutellar suture distinct. Scutellum almost flat, with fine lateral carinae. Metanotum with short, narrow and rounded median tooth. Subalar depression shallow and wide. Mesopleural pit deep, short and narrow. Sternaulus (precoxal suture) absent (Figs 1, 14). Prepectal carina shortly following on lateral sides of mesopleuron. Metapleural flange rather long, wide basally and narrowed towards apex, rounded apically. Propodeum without (Fig. 2) or with delineated areas, in first case with complete median and two lateral carinae; lateral tubercles absent. Propodeal spiracles rather small and round. Metapleural suture distinct. Metasternum medioventrally with distinct narrow and closely situated pair of tubercles.

Wings (Figs 12, 21). Pterostigma of fore wing narrow and long. Radial vein arising distinctly before middle of pterostigma. Recurrent vein weakly postfurcal. Nervulus almost interstitial or weakly postfurcal. Discoidal cell long and shortly petiolate anteriorly. Anterior abscissa of basal vein thickened. Basal and recurrent veins subparallel. Parallel vein arising from basal 0.15–0.2 of apical side of brachial cell. First abscissa of costal vein of hind wing about 0.5 times as long as second abscissa. Radial vein arising from costal vein closely to basal vein. Radial cell without additional transverse vein. Medial cell 10.0–11.0 times longer than width, 0.55–0.6 times as long as hind wing. Nervellus present. Submedial cell small. First abscissa of mediocubital vein 0.2–0.27 times as long as second abscissa. Recurrent vein short, weakly antefurcal, weakly oblique toward base of wing.

Legs. Fore coxa distinctly enlarged. Fore femur (Figs 9, 17) clavate, dorsally with keel in subapical 0.4, along inner margin in upper half with coarse and semi-circular numerous coarse carinae. Fore trochantellus dorsally with distinct transverse carina transformed in pointed flange on its inner part. Fore tibia with numerous and rather short thick spines arranged in almost single row (Figs 9, 17). Fore tarsus very long, more than 3.0 times longer than fore tibia, about 1.5 times longer than mesosoma. Middle tibia without spines. Middle trochantellus in outer apical part with distinct pointed tooth (Figs 10, 17). Hind coxa long and narrow, with distinct baso-ventral tooth in female (Figs 11, 14, 16) and without it in male (Fig. 10). Hind femur clavate (Fig. 16). Hind tibia with at least two spines on apical outer margin near spur, with patch of dense setae on inner apical part. Hind tibial spurs rather short, slender, setose, inner spur 0.17–0.25 times as long as hind basitarsus. Basitarsus of hind tarsus dorsally weakly concave, ventrally with narrow keel, 0.8–0.85 times as long as second-fifth segments combined. Claws short, with very wide and obtuse basal lobe.

Metasoma. First tergum long or very long, more or less narrow, petiolate (Figs 4–5, 18). Acrosternum 0.75–0.8 times as long as first tergum (Figs 3, 16). Basolateral lobes of first tergum absent; spiracles situated in basal 0.4–0.45 of tergum on large spiracular tubercles. Second tergum with short basal and almost triangular area separated shallow and distinctly convergent furrows, absent or almost absent posteriorly (Figs 6, 19). Second suture straight, shallow, sometimes partly almost indistinct. Second and third (at least basally) terga with separate lateroterga (Fig. 16). Hypopygyum of female rather large, pointed apically, upper subapically with pointed and rather narrow additional lobes (Fig. 20). Ovipositor with two obtuse and small dorsal nodes apically, densely serrate ventro-apically. Ovipositor sheath almost as long as body, with contrasting pale band in subapical 0.2–0.3.

#### Distribution.

Afrotropical Region

#### Key to species of the genus *Stephanospathius*

**Table:** 

1	Petiole narrow, its length 9.0–13.0 times maximum width, 1.1–1.25 times length of mesosoma (Figs 4, 5). Median length of second tergum 3.0–4.5 times its basal width. Mesoscutum of female medioposteriorly densely rugulose. Body length 10.6–16.2 mm. – Cameroon, Republic of Congo	*Stephanospathius ornatipes* (Kieffer)
–	Petiole wide, its length 4.3 times maximum width, 0.7 times length of mesosoma (Fig. 18). Median length of second tergum 1.6 times its basal width (Fig. 19). Mesoscutum of female medioposteriorly finely punctate and with distinct median carina. Body length 13.9 mm. – Republic of Congo	*Stephanospathius benoiti* sp. n.

### Stephanospathius ornatipes

(Kieffer, 1911)

http://species-id.net/wiki/Stephanospathius_ornatipes

Figs 1–12

Stenophasmus ornatipes Kieffer, 1911: 119.
Stephanospathius ornatipes : Belokobylskij 1992: 919, Yu et al. 2005.

#### Examined material.

Male (holotype), “Kamerun, Conradt”, “Syntypus” (red), “*Stenophasmus ornatipes* n. sp.” (handwriting by Kieffer), “Kieffer det.” (handwriting), “Holotypus *Stenophasmus ornatipes* Kieffer” (red), “*Stephanospathius ornatipes* (Kieffer), det. Belokobylskij, 1992” (SDEI); 1 female, Republic of Congo, “Musee du Conco, Mayumbe: Dinai, 19 – X – 1924, A. Collart” (MRAC); 1 female, Republic of Congo, “Musee du Conco, Mayumbe: Buhurumbe (?), 10 – X – 1924, A. Collart” (ZISP); 1 female (without head and most part of legs), Republic of Congo, “Musee du Conco, Eala, VII – 1936, J. Ghesquiere” (MRAC).

#### Description.

##### Female

Body length 10.6–16.2 mm; fore wing length 7.4–10.8 mm.

*Head* width 1.1–1.2 times its median length, 1.0–1.1 times its total length, 1.15–1.2 times its maximum height, 1.2–1.3 times maximum width of mesoscutum. Head behind eyes (dorsal view) weakly convex anteriorly and distinctly roundly narrowed posteriorly or entirely roundly narrowed. Transverse diameter of eye 1.4–1.5 times longer than temple. Ocelli medium-sized, in triangle with base 1.3–1.4 times its sides, situated before middle of head. POL 1.5 times Od, 0.6–0.7 times OOL. Occiput with dense and white setae (dorsal view). Eye 1.25–1.3 times as high as broad. Malar space height 0.2–0.25 times height of eye, 0.5–0.7 times basal width of mandible. Face width 0.8–0.85 times height of eye and 1.0–1.1 times height of face and clypeus combined. Clypeus distinctly convex. Width of hypoclypeal depression 1.1–1.3 times distance from edge of depression to eye, 0.45–0.5 times width of face. Hypostomal flange very narrow.

*Antennae* slender, weakly setiform, more than 54-segmented (apical segments missing). Scape short and wide, apically curvedly cutting along outer side, 1.5–1.6 times longer than maximum width. First flagellar segment almost round in dissection, distinctly curved basally, 5.0–5.5 times longer than its apical width, 0.9–0.95 times as long as second segment. Most flagellar segments with fine median constriction. Subapical segments 3.3 times longer than their width.

*Mesosoma* 2.7–3.2 times longer than its height, 2.6 times longer than its maximum width. Median lobe of mesoscutum distinctly protruding forward and convex anteriorly. Mesoscutum 1.3–1.4 times longer than its maximum width. Notauli finely and widely striate. Prescutellar depression shallow, rather narrow medially and wider laterally, with distinct median carina, almost smooth on rest part or sometimes with short sparse rugae, medially 0.2 times as long as scutellum. Scutellum flat, 1.1 times longer than maximum width. Subalar depression almost entirely finely rugulose-reticulate, with distinct and short striae anteriorly.

*Wings*. Fore wing 5.0–5.3 times longer than its maximum width. Pterostigma 6.0–7.0 times longer than wide. Metacarp 1.4–1.7 times longer than pterostigma. First and second radial abscissae forming very obtuse angle. Second radial abscissa 3.0–3.5 times longer than first abscissa, 0.55–0.6 times as long as the almost straight third abscissa, 1.3–1.5 times longer than first radiomedial vein. Second radiomedial cell long and wide, 3.2–3.6 times longer than its maximum width, 0.85–1.0 times as long as the rather wide brachial cell. First medial abscissa distinctly concavely curved. Nervulus postfurcal, distance from nervulus to basal vein about 0.4 times nervulus length. Hind wing 7.5–8.0 times longer than wide. First costal abscissa 0.45–0.5 times as long as second abscissa. Recurrent vein short, straight or weakly curved, weakly postfurcal or antefurcal, pigmented.

*Legs*. Hind coxa elongate-oval, with long and rather narrow basoventral tooth, 2.5 times without tooth or 2.2–2.3 times with tooth longer than wide, tooth with dense and white pubescence. Hind femur 4.8–5.1 times longer than wide, 0.55–0.6 times as long as hind tibia. Hind tarsus 1.1 times longer than hind tibia. Second segment of hind tarsus 0.5 times as long as basitarsus, 4.0 times longer than fourth segment, 2.0 times longer than fifth segment (without pretarsus).

*Metasoma* 1.8–2.0 times longer than head and mesosoma combined. Petiole very weakly or weakly and evenly widened towards apex. Maximum width of first tergum almost equal to its width at level of spiracular tubercles, 1.8–2.0 times its minimum width; length of petiole 7.3–8.8 times its maximum width, 0.9–1.1 times length of mesosoma, 0.7–0.85 times length of head and mesosoma combined. Median length of second tergum 2.5–3.0 times its basal width, 1.3–1.4 times length of third tergum. Length of second and third terga combined 5.0 times basal width of second tergum, 0.6–0.7 times length of petiole.

*Sculpture and pubescence*. Vertex, frons and temple smooth, temple below with distinct striation on narrow part; face almost entirely coarsely and densely rugose-reticulate, clypeus densely reticulate-rugose. Pronotum mostly smooth, its side below distinctly curvedly striate at narrow area. Mesoscutum smooth on median lobe, its lateral lobes finely and densely punctate, medially mesoscutum densely rugulose or rugulose-striate. Scutellum almost smooth or sparsely and finely punctate. Mesopleuron smooth in lower 0.8–0.9. Propodeum without delineated areas, with distinct and complete median and two weakly curvedly convergent posteriorly lateral longitudinal carinae, entirely coarsely and mostly transversely rugose-striate, sculpture medially sometimes distinctly fine. Metapleuron rugose-striate, but sometimes sculpture fine. Hind coxae densely transversely striate in dorsal half, finely rugulose-striate to almost smooth in ventral half. Hind femur dorsally very finely rugulose-reticulate, sometimes almost smooth, smooth on the most part. First tergum almost entirely densely transversely striate, often finely sculptured basally and almost smooth apically at short distance. Second tergum mostly smooth, rugose-striate in basal area. Remaining terga smooth. Vertex in posterior 0.2–0.3 with rather dense, long and semi-erect pale setae, remainder of vertex glabrous. Median lobe of mesoscutum sparsely and shortly setose, almost glabrous anteriorly; lateral lobes entirely with dense, semi-erect and mainly short pale setae, with additional sparse long setae along notauli and marginally. Metapleural lobe with short and dense pubescence. Hind tibia dorsally with numerous and dense short and sparse long semi-erect setae; length of long setae 0.5–0.9 times maximum width of hind tibia.

*Colour*. Head reddish brown or dark reddish brown, darkened dorsally, face sometimes mostly yellowish brown, malar space yellow or brownish yellow. Metasoma almost black. Petiole and second metasomal tergum reddish brown or dark reddish brown, often petiole black, remaining terga brownish yellow or light reddish brown, but infuscate medio-posteriorly. Palpi pale yellow. Fore and middle legs yellow or pale brownish yellow, fore tarsi infuscate, middle trochantellus and wide submedian part of femur more or less brown. Hind coxa, trochanter, trochantellus and femur dark reddish brown to almost black at least partly, tibia brownish yellow, but infuscate apically, tarsus yellowish brown; apical segments of all tarsi infuscate. Fore wing evenly and very faintly infuscate. Pterostigma entirely dark brown.

##### Male

Body length 11.6 mm; fore wing length 6.6 mm.

*Head* width 1.1 times its median length, equal to its total length, 1.15 times its maximum height, equal to maximum width of mesoscutum. Head behind eyes (dorsal view) weakly convex anteriorly and distinctly roundly narrowed posteriorly. Transverse diameter of eye 1.3 times longer than temple. Ocelli medium-sized, in triangle with base 1.3 times its sides, situated before middle of head. POL 1.4 times Od, 0.75 times OOL. Occiput with very dense and white setae (dorsal view). Eye 1.3 times as high as broad. Malar space height 0.2 times height of eye, about 0.5 times basal width of mandible. Face width 0.85 times height of eye and 1.1 times height of face and clypeus combined. Clypeus distinctly convex. Width of hypoclypeal depression equal to distance from edge of depression to eye, 0.4 times width of face. Hypostomal flange very narrow.

*Antennae* entirely missing.

*Mesosoma* 3.2 times longer than its height, about 3.0 times longer than its maximum width. Median lobe of mesoscutum distinctly protruding forward and convex anteriorly. Mesoscutum 1.4 times longer than maximum width. Notauli mostly almost smooth. Prescutellar depression shallow, rather narrow medially and wider laterally, with distinct median carina, almost entirely smooth, medially 0.2 times as long as scutellum. Scutellum flat, 1.1 times longer than maximum width. Subalar depression entirely rugose-striate.

*Wings*. Fore wing 4.8 times longer than its maximum width. Pterostigma 6.5 times longer than width. Metacarp 1.3 times longer than pterostigma. First and second radial abscissae forming very obtuse angle. Second radial abscissa 4.0 times longer than first abscissa, 0.6 times as long as the straight third abscissa, 1.5 times longer than first radiomedial vein. Second radiomedial cell long and wide, 3.3 times longer than its maximum width, 0.9 times as long as the wide brachial cell. First medial abscissa distinctly curved. Nervulus very weakly postfurcal. Hind wing 7.0 times longer than width. First costal abscissa 0.5 times as long as second abscissa. Recurrent vein short, straight, weakly postfurcal, pigmented.

*Legs*. Hind coxa without basoventral tooth, elongate-oval, 2.7 times longer than width. Hind femur 4.8 times longer than width, 0.55 times as long as hind tibia. Hind tarsus almost as long as hind tibia. Second segment of hind tarsus 0.45 times as long as basitarsus, 3.3 times longer than fourth segment, 1.6 times longer than fifth segment (without pretarsus).

*Metasoma* 2.4 times longer than head and mesosoma combined. Petiole mostly almost parallel-sided, weakly and curvedly widened in apical 0.25. Maximum width of first tergum 0.85 times its width at level of spiracular tubercles, 1.6 times its minimum width; length of petiole 13.0 times its maximum width, 1.25 times length of mesosoma, 0.95 times length of head and mesosoma combined. Median length of second tergum 4.5 times its basal width, 1.4 times length of third tergum. Length of second and third terga combined almost 8.0 times basal width of second tergum, 0.65 times length of petiole

*Sculpture and pubescence*. Vertex, frons and temple smooth; face almost entirely coarsely and densely striate-rugose, clypeus rugose. Pronotum mostly smooth, its side below distinctly striate at narrow area. Mesoscutum mostly smooth, its lateral lobes finely punctate, finely rugulose latero-marginally. Scutellum smooth. Mesopleuron smooth in lower 0.7. Propodeum without delineated areas, with distinct median and two weakly convergent posteriorly lateral longitudinal complete carinae, entirely coarsely and mostly transversely rugose-striate. Metapleuron coarsely rugose-striate. Hind coxae entirely densely transversely and semi-circularly striate. Hind femur dorsally finely rugulose-reticulate. First tergum entirely densely transversely striate, sometimes partly rugulose-striate. Second tergum basally finely and densely striate. Remaining terga smooth. Vertex in posterior 0.3 with rather dense, long and semi-erect pale setae, remainder of vertex glabrous. Median lobe of mesoscutum sparsely and shortly setose, glabrous anteriorly; lateral lobes almost entirely with dense, semi-erect and mainly short pale setae, with additional sparse long setae along notauli and marginally. Metapleural lobe with short and dense pubescence posteriorly. Hind tibia dorsally with numerous and dense short and sparse long semi-erect setae; length of long setae 0.4–0.7 times maximum width of hind tibia.

*Colour*. Head and metasoma reddish brown to dark reddish brown, face laterally pale, malar space yellow. Petiole yellowish brown, remainder metasoma brownish yellow, but brown apically. Palpi pale yellow. Fore and middle legs yellow or brownish yellow. Hind coxa and trochanter reddish brown, femur brownish yellow, tibia and tarsus almost yellow; apical segments of all tarsi infuscate. Fore wing hyaline. Pterostigma entirely brown.

### Stephanospathius benoiti sp. n.

urn:lsid:zoobank.org:act:EBC1B03B-90B0-46DF-9B23-36D6090F8782

http://species-id.net/wiki/Stephanospathius_benoiti

Figs 13–21

#### Type material.

Holotype: female, Republic of Congo, “Museé du Congo, Eala, VII–1936, J. Chesquiere”, “R. dét. cc. 4917” (MRAC).

#### Description.

##### Female

Body length 13.9 mm; fore wing length 9.4 mm.

*Head* width 1.3 times its median length, 1.1 times its total length, 1.15 times its maximum height, 1.2 times maximum width of mesoscutum. Head behind eyes (dorsal view) roundly narrowed. Transverse diameter of eye 1.25 times longer than temple. Ocelli medium-sized, in triangle with base 1.3 times its sides, situated almost on middle of head. POL 1.4 times Od, 0.6 times OOL. Occiput with very dense and white setae (dorsal view). Eye 1.3 times as high as broad. Malar space height 0.2 times height of eye, 0.5 times basal width of mandible. Face width 0.85 times height of eye and 1.1 times height of face and clypeus combined. Clypeus distinctly convex. Width of hypoclypeal depression equal to distance from edge of depression to eye, 0.45 times width of face. Hypostomal flange very narrow.

*Antennae* slender, weakly setiform, more than 62-segmented (apical segments missing). Scape short and wide, apically curvedly cutting along outer side, 1.5 times longer than maximum width. First flagellar segment weakly curved basally, 4.5 times longer than apical width, almost as long as second segment. Most flagellar segments with fine median constriction. Subapical segments about 3.5 times longer than their width.

*Mesosoma* 2.9 times longer than its height, 2.7 times longer than its maximum width. Median lobe of mesoscutum distinctly protruding forward and convex anteriorly. Mesoscutum 1.35 times longer than maximum width. Notauli finely striate anteriorly, but mostly smooth. Prescutellar depression shallow, rather narrow medially and wider laterally, medially 0.15 times as long as scutellum, with distinct median carina, smooth. Scutellum flat, 1.1 times longer than maximum width. Subalar depression widely finely and densely punctate, with sparse short striation along upper side.

*Wings*. Fore wing 5.1 times longer than its maximum width. Pterostigma about 5.5 times longer than wide. Metacarp 1.5 times longer than pterostigma. First and second radial abscissae forming very obtuse angle. Second radial abscissa 3.3 times longer than first abscissa, 0.6 times as long as the almost straight third abscissa, 1.6 times longer than first radiomedial vein. Second radiomedial cell long and wide, 3.1 times longer than its maximum width, 0.7 times as long as the wide brachial cell. First medial abscissa distinctly concavely curved. Nervulus weakly postfurcal, distance from nervulus to basal vein 0.1–0.2 times nervulus length. Hind wing 7.0 times longer than wide. First costal abscissa 0.5 times as long as second abscissa. Recurrent vein short, straight, weakly postfurcal, pigmented.

*Legs*. Hind coxa with long and rather narrow basoventral tooth, elongate-oval, 2.4 times without tooth and 2.2 times with tooth longer than wide, tooth with dense and white pubescence apically. Hind femur 4.7 times longer than wide, 0.65 times as long as hind tibia. Hind tarsus 1.1 times longer than hind tibia. Second segment of hind tarsus 0.5 times as long as basitarsus, 3.5 times longer than fourth segment, 1.8 times longer than fifth segment (without pretarsus).

*Metasoma* 1.6 times longer than head and mesosoma combined. Petiole weakly, but distinctly and rather evenly widened towards apex. Maximum width of first tergum 1.1 times its width at level of spiracular tubercles, 2.1 times its minimum width; length of petiole 4.3 times its maximum width, 0.7 times length of mesosoma, 0.55 times length of head and mesosoma combined. Median length of second tergum 1.6 times its basal width, 1.1 times length of third tergum. Length of second and third terga combined 3.3 times basal width of second tergum, 0.9 times length of petiole. Ovipositor sheath 0.9 times as long as body.

*Sculpture and pubescence.* Vertex, frons and most part of temple smooth, temple below and posteriorly curvedly striate; face entirely coarsely and densely rugose-reticulate with granulation. Pronotum mostly smooth, its side below distinctly and curvedly striate at narrow area. Mesoscutum smooth on median lobe, but finely punctate in posterior half and with rather distinct median carina, its lateral lobes finely and densely punctate. Scutellum very finely and densely punctate. Mesopleuron mostly smooth. Propodeum with large basolateral areas weakly delineated by carinae, medial carina 0.7 times as long as propodeum medially; propodeum almost entirely distinctly and mostly transversely rugose-striate, basolateral areas medially almost smooth on narrow area. Metapleuron rugose-striate, sparsely punctate with smooth areas in anterior 0.3. Hind coxae densely transversely striate with fine granulation in dorsal half, finely striate to almost smooth in ventral half. Hind femur dorsally very finely striate and partly with punctation. First tergum coarsely rugose-striate in basal 0.4, curvedly and transversely densely striate and partly with fine granulation in apical 0.6. Second tergum mostly very finely striate-coriaceous, coarse rugose-striate in small basal area. Remaining terga smooth. Vertex in posterior 0.2–0.3 and temple with rather dense, long and semi-erect pale setae, remainder of vertex glabrous. Median lobe of mesoscutum sparsely and shortly setose, nut much dense along notauli, lateral lobes entirely with dense, semi-erect and mainly short pale setae, with a few long setae along notauli. Metapleural lobe posteriorly with short and dence pubescence. Hind tibia dorsally with numerous and dense short and sparse long semi-erect setae; length of long setae 0.4–0.8 times maximum width of hind tibia.

*Colour*. Head black, face light reddish brown, darkened dorsally, malar space yellowish brown. Metasoma almost black. Petiole black, second metasomal tergum dark reddish brown, remaining terga reddish brown with dark places, brownish yellow or yellow laterally and below. Palpi yellow or pale yellow. Fore leg light reddish brown, fore tibia darkened basally; middle coxa, trochanters and most part of femur dark reddish brown, apical 0.4 of femur, tibia and tarsus light reddish brown. Hind coxa, trochanters and femur black, hind tibia dark reddish brown, tarsus reddish brown. Fore wing evenly and faintly infuscate. Pterostigma entirely dark brown.

#### Diagnosis.

This new species resembles *Stephanospathius ornatipes*; the main differences between these taxa are indicated in the key.

#### Etymology.

In honour of Dr. P.L.G. Benoit, who firstly recognised this new species.

**Figures 13–21.**
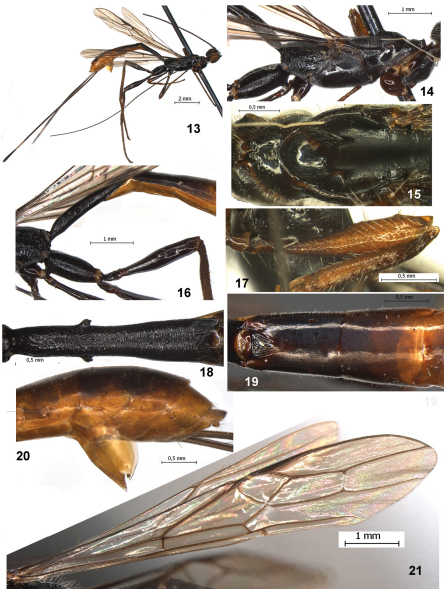
*Stephanospathius benoiti* sp. n. female **13** total view **14** mesosoma and coxae, lateral view **15** pronotum and mesoscutum **16** propodeum, hind coxa and femur and basal half of metasoma, lateral view **17** fore femur and tibia, inner view **18** first tergite, dorsal view **19** second and third tergites, dorsal view **20** apical part of metasoma with hypopygium, lateral view **21** fore and hind wings.

## Discussion

Subfamily Stephaniscinae was erected by Enderlein (1912) for eight doryctine genera with type genus *Stephaniscus* Kieffer. Ten mostly not closely related genera were included in the tribe Stephaniscini in Braconidae Catalogue by Shenefelt and Marsh (1976). The status and composition of this tribe was reassessed by Belokobylskij (1992) in his reclassification of subfamily Doryctinae, which suggested a new apomorphic character of Stephaniscini (the presence of propodeal bridge) and substantially reduced the number of genera to three (*Schlettereriella* Szepligeti, *Leptospathius* Szepligeti, *Stephanospathius* Belokobylskij). Later Wharton and Achterberg (2000) and Yu et al. (2005) showed that this tribal name is “invalid because its type genus *Stephaniscus* Kieffer, 1904 is a junior homonym of *Stephaniscus* Haeckel, 1884”. As a result, in the last World Catalogue (Yu et al. 2005), the members of the tribe Stephaniscini were placed in the tribe Spathiini. However, I have retained this tribe under the substitutional name Leptospathiini nom. n. that includes five genera (*Austrospathius* Belokobylskij, Iqbal & Austin, *Leptospathius* Szepligeti, *Oroceguera* Seltmann & Sharkey, *Schlettereriella* Szepligeti and *Stephanospathius* Belokobylskij) because the diagnostic characters of this group are important and evolutionally valuable (Belokobylskij 1992).

Tribe Leptospathiini is related to some members of the tribe Spathiini (which requires reclassification: see, for example, Zaldivar-Riveron et al. 2008). Both of these tribes are characterised by the petiolate first metasomal tergum with its acrosternum distinctly or strongly elongated. However, the elongated acrosternum appears several times in different phylogenetic lines of subfamily Doryctinae (Belokobylskij 2007, Zaldivar-Riveron et al. 2007), and a recent molecular phylogenetic analysis showed that the genera of the Neotropical Spathiini genera formed a separate cluster of taxa in comparison with Old World Spathiini genera (Zaldivar-Riveron et al. 2008). The acrosternum is very long (larger than a half of tergum) in the members of the tribe Spathiini. On the other hand, the members of Leptospathiini have significant variation of the acrosternum length from weakly elongated (less then a half of tergum in *Leptospathius*, *Schlettereriella* and *Oroceguera*) to strongly elongated (more than half of tergum in *Austrospathius* and *Stephanospathius*). The main apomorphic character of Leptospathiini is the presence of a distinct and usually wide propodeal bridge (heavy sclerites between coxal and metasomal foramens). This feature is unique for Doryctinae taxa and undoubtedly evolutionally significant.

According to current information on the internal structures of doryctine ovipositors (Rahman et al. 1998), *Leptospathius* and *Schlettereriella* are closely related (and distinctly separate from *Spathius* Nees) particularly in characters such as a loss of the valvillus and the modification of the most anterior subctenidial seta into a branched “pseudovalvillus”. These characters are not recorded in *Stephanospathius* (and unknown for *Austrospathius* and *Oroceguera*) and this genus has developed valvillus and normal subctenidial seta, which were the additional reason (besides external morphology) to place this genus separately in the tribe. The structures of the venom glands and reservoir of *Leptospathius* and *Schlettereriella* are also very similar (Quicke et al. 1992), but do not have principal differences with other doryctine taxa (information about these structures in *Stephanospathius* is absent).

Similar result was obtained in Zaldivar-Riveron et al.’s (2008) molecular phylogeny of the Doryctinae genera, although *Leptospathius* was not included in the study. The position of *Stephanospathius* on the phylogenetic trees is highly variable and different in every tree, but in only a single tree does this genus have subbasal nesting with Old World *Spathius* taxa. On the other hand, *Schlettereriella* in all phylogenetic trees was a sister group to African *Pseudodoryctes* Szepligeti, *Doryctoproctus* Belokobylskij and *Rinamba* Cameron in an as yet unresolved cluster of African-Holarctic doryctine genera. These ambivalent results undoubtedly show that only an additional wide and comprehensive morphological and molecular study of Leptospathiini and related genera can help us to understand the real status and phylogeny of this peculiar tribe.

## Supplementary Material

XML Treatment for Leptospathiini

XML Treatment for Stephanospathius

XML Treatment for Stephanospathius ornatipes

XML Treatment for Stephanospathius benoiti
